# Metformin Prevents Tumor Cell Growth and Invasion of Human Hormone Receptor-Positive Breast Cancer (HR+ BC) Cells via *FOXA1* Inhibition

**DOI:** 10.3390/ijms25137494

**Published:** 2024-07-08

**Authors:** Christine Song, Dawa Jung, Ayse Tuba Kendi, Jin Kyung Rho, Eun-Joo Kim, Ian Horn, Geoffry L. Curran, Sujala Ghattamaneni, Ji Yeon Shim, Pil Soo Kang, Daehun Kang, Jay B. Thakkar, Sannidhi Dewan, Val J. Lowe, Seung Baek Lee

**Affiliations:** 1 Division of Radiology, Mayo Clinic, Rochester, MN 55905, USA; csong@college.harvard.edu (C.S.); jung.dawa@mayo.edu (D.J.); kendi.ayse@mayo.edu (A.T.K.); horn.lan@mayo.edu (I.H.); curran.geoffry@mayo.edu (G.L.C.); ghattamaneni.sujala@mayo.edu (S.G.); kang.daehun@mayo.edu (D.K.); jay.thakkar@jefferson.edu (J.B.T.); sannidhi.dewan@jefferson.edu (S.D.); 2 Harvard University, Cambridge, MA 02138, USA; 3 Department of Convergence Medicine, University of Ulsan College of Medicine, Seoul 05505, Republic of Korea; jkrho@amc.seoul.kr; 4 Department of Molecular Biology, Dankook University, Cheonan 31116, Chungcheongnam, Republic of Korea; nbrejk@dankook.ac.kr; 5 College of Nursing, Dankook University, Cheonan 31116, Chungcheongnam, Republic of Korea; modern0270@gmail.com; 6 U&Hang Clinic, Asan 31514, Chungcheongnam, Republic of Korea; analgesics@naver.com; 7 Department of Molecular Pharmacology and Experimental Therapeutics, Mayo Clinic, Rochester, MN 55905, USA

**Keywords:** hormone-receptor-positive (HR+) breast cancer (BC), type 2 diabetes (T2D), forkhead box A1 (*FOXA1*), metformin, tumor proliferation, metastasis

## Abstract

Women with type 2 diabetes (T2D) have a higher risk of being diagnosed with breast cancer and have worse survival than non-diabetic women if they do develop breast cancer. However, more research is needed to elucidate the biological underpinnings of these relationships. Here, we found that forkhead box A1 (*FOXA1*), a forkhead family transcription factor, and metformin (1,1-dimethylbiguanide hydrochloride), a medication used to treat T2D, may impact hormone-receptor-positive (HR+) breast cancer (BC) tumor cell growth and metastasis. Indeed, fourteen diabetes-associated genes are highly expressed in only three HR+ breast cancer cell lines but not the other subtypes utilizing a 53,805 gene database obtained from NCBI GEO. Among the diabetes-related genes, *FOXA1*, *MTA3*, *PAK4*, *FGFR3*, and *KIF22* were highly expressed in HR+ breast cancer from 4032 breast cancer patient tissue samples using the Breast Cancer Gene Expression Omnibus. Notably, elevated *FOXA1* expression correlated with poorer overall survival in patients with estrogen-receptor-positive/progesterone-receptor-positive (ER+/PR+) breast cancer. Furthermore, experiments demonstrated that loss of the *FOXA1* gene inhibited tumor proliferation and invasion in vitro using MCF-7 and T47D HR+ breast cancer cell lines. Metformin, an anti-diabetic medication, significantly suppressed tumor cell growth in MCF-7 cells. Additionally, either metformin treatment or *FOXA1* gene deletion enhanced tamoxifen-induced tumor growth inhibition in HR+ breast cancer cell lines within an ex vivo three-dimensional (3D) organoid model. Therefore, the diabetes-related medicine metformin and *FOXA1* gene inhibition might be a new treatment for patients with HR+ breast cancer when combined with tamoxifen, an endocrine therapy.

## 1. Introduction

Breast cancer is one of the most commonly diagnosed cancers worldwide and the leading cause of cancer death [1,2,3,4]. Breast cancer can be categorized into three primary subtypes, hormone-receptor-positive (HR+ BC), HER2-positive (HER2+ BC), and triple-negative (TNBC), based on the presence or absence of the estrogen receptor (ER), progesterone receptor (PR), and ERBB2 (commonly known as HER2) [1,2,4]. Specifically, hormone-receptor-positive (HR+) breast cancer (BC) makes up approximately 60% to 75% of breast cancer patients, and the five-year survival rate of stage IV ER+/PR+ breast cancer patients is below 20% [4,5]. Endocrine therapy is the mainstay of treatment for HR+ BC, significantly reducing the recurrence rate for stages 1–3 [6]. Despite recent advances in endocrine therapy, many women unfortunately relapse during or after the completion of adjuvant therapy [6,7]. Alterations in the PI3K/AKT/mTOR pathway are prevalent in hormonal breast cancer, over-activating the pathway and allowing uncontrolled tumor cell growth, resistance to apoptosis, and tumorigenesis [8,9]. Therefore, inhibitors of the PI3K/AKT/mTOR pathway are an important part of the current clinical management of HR+ metastatic breast cancer [8,9]. One of the common endocrine therapy options for the treatment of HR+ BC is tamoxifen [10,11]. Tamoxifen was approved by the Food and Drug Administration (FDA) in 1977 for women with advanced HR+ breast cancer (BC) and a few years later as adjuvant treatment for women with early-stage breast cancer [10]. Tamoxifen is well known as a groundbreaking adjuvant endocrine therapy that blocks the binding of estrogen to the estrogen receptor, preventing the ERα protein from reaching the transcription stage [1,6,10,12]. The great advantage of this hormone treatment is that it can significantly reduce the cancer recurrence rate to 50% when administered for 5 years, thereby improving the patient survival rate to 60%. However, even with this treatment, there are many cases where people stop taking the drug due to facial flushing, uterine cancer, blood clots, as well as gastrointestinal hypersensitivity, headache, and dizziness [1,3,4,5,6,10,13,14]. Therefore, it is critical to find new treatments that minimize side effects while enhancing the efficacy against HR+ BC.

Diabetes mellitus is a serious and common disease that happens when insulin is not secreted by the pancreatic beta cells or when systemic cells are insulin resistant [11,12,15,16]. According to the International Diabetes Federation (IDF), as of 2015, approximately 415 million adults aged 20 to 79 had diabetes [11]. While type 1 diabetes (T1D) is caused by an autoimmune disease that results in the absence of insulin secretion, type 2 diabetes (T2D) is caused by both the resistance and inadequate secretory response of insulin [11,12,15,16]. Several current papers report that women have a 23% increased risk of developing breast cancer when they have T2D compared to non-diabetic females [17,18]. Moreover, breast cancer patients with T2D have a 50% higher risk of death than non-diabetic breast cancer patients [19]. However, the specific subtype of breast cancer that is most increased in the setting of T2D and the mechanism underlying the relationship between T2D and breast cancer is unclear. Metformin (1,1-dimethylbiguanide hydrochloride) is a common oral medication for T2D patients. Mechanistically, metformin activates the AMP-activated protein kinase (AMPK), which blocks the mTOR signal to inhibit cancer cell growth and invasion [20,21]. Nonetheless, there is a paucity of data about the genes that metformin regulates in HR+ breast cancer specifically.

Forkhead box A1 (*FOXA1*) is a transcription regulator and is the first member of the FOX family that contains at least 40 members [22,23,24]. FOXA1 binding to nucleosomes regulates an open chromatin configuration, enabling the recruitment of other transcriptional regulators [23,24]. It also contributes to chromatin remodeling by binding to DNA, which precedes the loss of cytosine methylation and the demethylation of histone H3 lysine (H3K4) [25]. FOXA1 is expressed and regulates development from the early stages to adulthood [24]. It also has a critical role in glucose homeostasis regulation through α-islet cell function directing. Deletion of *FOXA1* causes the loss of insulin in response to glucose administration in mice as well as decreasing glucagon levels [22,23,24]. Several papers suggest the role of FOXA1 in various cancers, such as breast cancer, acute myeloid leukemia (AML), esophageal cancer, lung cancer, pancreatic cancer, and thyroid cancer [23,24,25]. *FOXA1* is highly amplified in AML patient samples and amplified in thyroid cancer. Furthermore, FOXA1 expression affects lymph node metastasis in esophageal squamous-cell carcinoma (ESCC), and the knockdown of *FOXA1* decreases cell migration and invasion in vitro [24]. While some studies suggest that *FOXA1* is highly upregulated in HR+ BC, other papers report the high expression of *FOXA1* in TNBC [22,23,24].

In this study, we attempted to find breast cancer subtypes that were associated with T2D using the 53,805 gene expression data set from NCBI GEO. We confirmed that 14 related genes found in three different human HR+ breast cancer groups were associated with T2D. These diabetes-related genes, using Breast Cancer Gene Expression data, showed high gene expression and low survival rates in HR+ BC but not TNBC. We also performed several experiments to determine if the diabetes-related gene *FOXA1* plays an important role in tumor cell growth and invasion in human HR+ BC cell lines. We assessed the potential benefits of metformin, a diabetes treatment, in HR+ BC. In addition, we performed several experiments with the diabetes-related gene *FOXA1* to assess its role in tumor cell growth and invasion in human HR+ BC cell lines.

## 2. Results

### 2.1. Fourteen Diabetes-Related Genes Are Significantly Expressed in Hormone-Receptor-Positive Breast Cancer (HR+ BC) Cell Lines from the NCBI GEO Dataset

**Figure 1:**
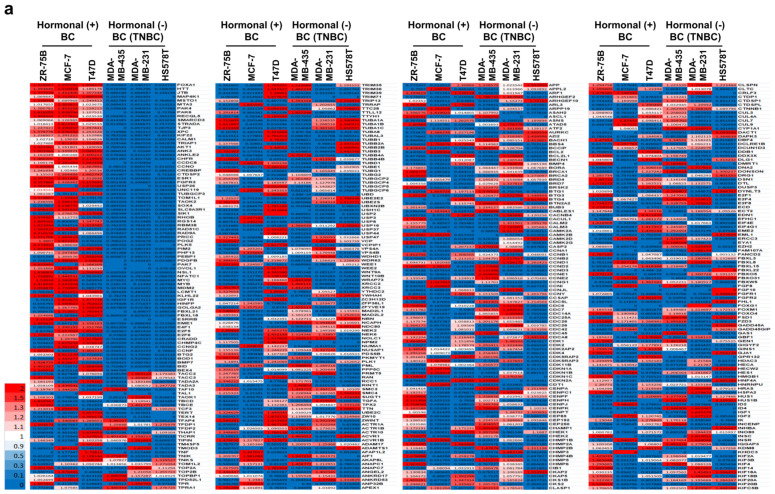

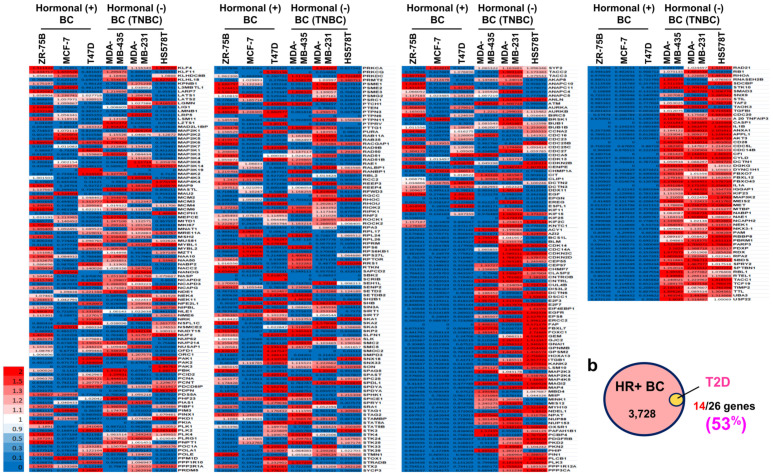
Hormone-receptor-positive breast cancer (HR+ BC) exhibits significantly higher expression of diabetes-related cell metabolism genes compared to triple-negative breast cancer (TNBC). (**a**) A total of 53,805 genes were analyzed from six breast cancer cell lines, including three TNBC lines (MDA-MB-231, MDA-MB-435, and HS578T) and three other breast cancer lines (ZR-75B, MCF-7, and T47D). The unique expression values for each gene were normalized to the housekeeping gene β-actin. Genes showing differential expression values of 1.5-fold or greater (*p* > 0.005) were identified, with upregulation indicated in red and downregulation in blue. (**b**) The gene groups overexpressed in hormone-receptor-positive breast cancer were investigated for their relationship with diabetes-related cell metabolism by synthesizing previously reported research papers and statistically analyzed.

We hypothesized that type 2 diabetes (T2D) might be related to a specific breast cancer subtype rather than to all breast cancer. To study which breast cancer subtype correlates with T2D, we assessed 53,805 genes and expressions in three different HR+ breast cancer cell lines, ZR-75B, MCF7, and T47D, and three TNBC cell lines, MDA-MB435, MDA-MB-231, and HS578T from the National Center for Biotechnology Information Gene Expression Omnibus (NCBI GEO). In a previous study, we found that HR+ breast cancer-related genes were related to cell metabolism, neuronal, and cell proliferation, while TNBC-related genes were related to cell metastasis and invasion, as well as inflammation [26,27,28]. Therefore, we hypothesized that HR+ breast cancer would be linked to T2D. We selected 26 genes (*KIF22*, *CALM1*, *TRIAP1*, *AKT1*, *ANK3*, *ANKLE2*, *CHFR*, *CCDC8*, *CCNO*, *CREBBP*, *CTDSP2*, *ESR1*, *FGFR3*, *FOXA1*, *HTT*, *JTB*, *MAP4K1*, *MSTO1*, *MTA3*, *PAK4*, *PAK6*, *RECQL5*, *SMARCD3*, *STRADA*, *TJP3*, and *XPC*) from 3728 HR+ breast cancer-related genes, focusing on those that were highly expressed in only the three HR+ breast cancer cell lines (Figure 1a). The genes were not detected in the TNBC cell lines. Among the 26 genes, 14 (*KIF22*, *CALM1*, *TRIAP1*, *AKT1*, *CHFR*, *CREBBP*, *ESR1*, *FGFR3*, *FOXA1*, *MAP4K1*, *MTA3*, *PAK4*, *PAK6*, and *SMARCD3*) genes were previously reported to be related to diabetes [29,30,31,32,33,34,35,36,37,38,39,40,41,42,43] (Figure 1b). A total of 53% of the 26 hormone-receptor-positive breast cancer (HR+ BC)-related genes were linked to T2D, suggesting that T2D has a strong relation to only HR+ BC but no other subtype BC.

### 2.2. Diabetes-Related Genes Are Highly Expressed in Hormone-Receptor-Positive Breast Cancer (HR+ BC) Patients

**Figure 2:**
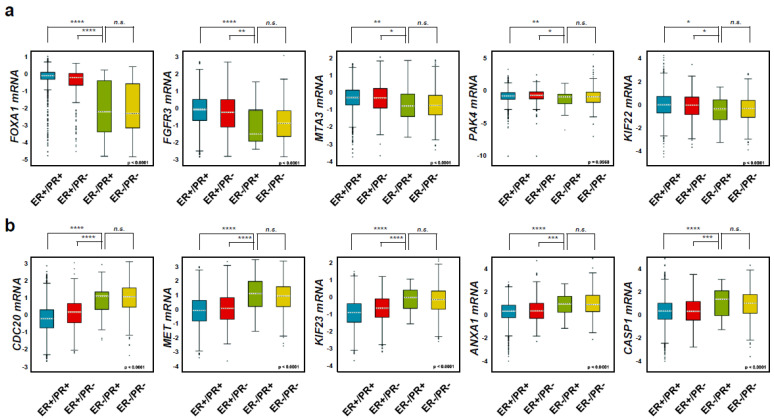
Diabetes-related genes are highly expressed in hormone-receptor-positive breast cancer (HR+ BC) patients. (**a**) Five diabetes-related genes (*FOXA1*, *FGFR3*, *MTA3*, *PAK4*, and *KIF22)* expressions were analyzed with 4032 patient data samples from the Breast Cancer Gene Expression Omnibus: ER+/PR+ (*n* = 3262), ER+/PR− (*n* = 283), ER−/PR+ (*n* = 45), ER−/PR− (*n* = 462). The center is the median, and the minimum and maximum values are represented by the whiskers. *p*-values were calculated using Welch’s test. * *p* < 0.05, ** *p* < 0.01, *** *p* < 0.001, and **** *p* < 0.0001; n.s., non-significant. (**b**) Five triple-negative breast cancer (TNBC)-related genes (*CDC20*, *MET*, *KIF23*, *ANXA1*, and *CASP1)* expressions were also checked through the Breast Cancer Gene Expression Omnibus as a negative control: ER+/PR+ (*n* = 3262), ER+/PR− (*n* = 283), ER−/PR+ (*n* = 45), ER−/PR− (*n* = 462). The center is the median, and the minimum and maximum values are represented by the whiskers. *p*-values were calculated using Welch’s test. * *p* < 0.05, ** *p* < 0.01, *** *p* < 0.001, and **** *p* < 0.0001; n.s., non-significant. ER, estrogen receptor; PR, progesterone receptor.

**Figure 3:**
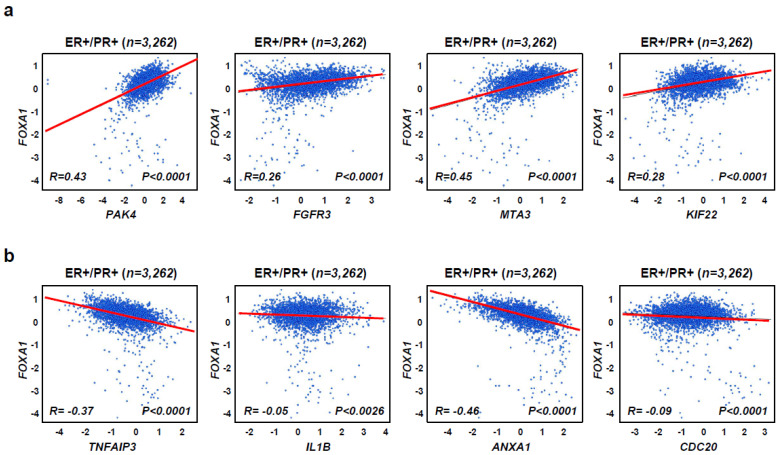
*FOXA1* and other diabetes-related genes show a positive correlation in ER+/PR+ breast cancer patients. (**a**) The correlation between *FOXA1* and diabetes-related genes (*PAK4*, *FGFR3*, *MTA3*, and *KIF22*) through the analysis of 3262 ER+/PR+ breast cancer patients from the Breast Cancer Gene Expression Omnibus. *p*-values were calculated using the Pearson correlation coefficient; *p* < 0.0001. (**b**) The correlation between *FOXA1* and TNBC-related genes (*TNFAIP3*, *IL1B*, *ANXA1*, and *CDC20*) in ER+/PR+ breast cancer was checked through the Breast Cancer Gene Expression Omnibus as a negative control. *p*-values were calculated using unpaired two-tailed Student’s *t*-tests; *p* < 0.0001 and *p* < 0.0026. ER, estrogen receptor; PR, progesterone receptor.

To examine the diabetes-related gene expression in different breast cancer subtypes, we used a dataset with four differentiated breast cancer subtypes (ER+/PR+, ER+/PR−, ER−/PR+, and ER−/PR−) with 4032 patient tissue samples from the Breast Cancer Gene Expression Miner v4.5 [26,28]. The newly identified diabetes-related gene, *FOXA1* [38] was highly amplified in ER+/PR+ (*n* = 3262) and ER+/PR− (*n* = 283) but not ER−/PR+ (*n* = 45) and ER−/PR− (*n* = 462) (Figure 2a). Other diabetes-related genes, such as *FGFR3* [29], *MTA3* [40], *PAK4* [31], and *KIF22* [43], had similar results. Collectively, these data confirm the association between T2D and HR+ breast cancer. However, five genes (*CDC20*, *MET*, *KIF23*, *ANXA1*, and *CASP1*) that are found in the TNBC group were not highly expressed in the HR+ breast cancer subtypes but are commonly found in the ER−/PR+ and ER−/PR− groups such that they could be used as negative controls [26,28] (Figure 2b). As *FOXA1* had the most significant gene expression, we checked the correlation between *FOXA1* and other diabetes-related genes such as *PAK4*, *FGFR3*, *MTA3*, and *KIF22* using a database containing 3262 ER+/PR+ breast cancer patient tissue samples (Figure 3). *FOXA1* expression was positively correlated to the four other diabetes-related genes, *PAK4*, *FGFR3*, *MTA3*, and *KIF22*, found in the HR+ breast cancer, but not to the genes highly expressed in TNBC such as *TNFAIP3*, *IL1B*, *ANXA1*, and *CDC20.* This suggests that *FOXA1* has a positive correlation to other diabetes-related genes in HR+ breast cancer (Figure 3a,b). Collectively, this indicates that genes highly expressed in a hormone-receptor-positive breast cancer (HR+ BC) subtype are closely connected to T2D.

### 2.3. High FOXA1 Expression Leads to Worse Overall Survival in Hormone-Receptor-Positive Breast Cancer (HR+ BC) Patients

**Figure 4:**
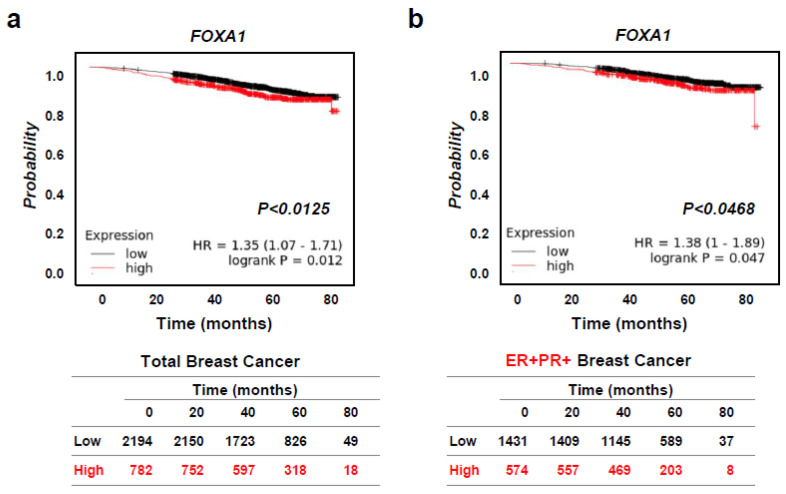
High *FOXA1* expression correlates to worse overall hormone-receptor-positive breast cancer (HR+ BC) patient survival. (**a**) Patient survival data were analyzed through the analysis of 2976 total breast cancer patients (low: *n* = 2194; high: *n* = 782) from the Kaplan–Meier plotter. *p*-values were calculated using unpaired two-tailed Student’s *t*-tests; *p* < 0.0125. (**b**) Patient survival data were analyzed through the analysis of 2976 ER+/PR+ breast cancer patients (low: *n* = 1431; high: *n* = 574) from the Kaplan–Meier plotter. *p*-values were calculated using unpaired two-tailed Student’s *t*-tests; *p* < 0.0468.

As there was an increased expression of the *FOXA1* gene expression was more in the HR+ breast cancer subtype than other diabetes-related genes, we focused on whether or not *FOXA1* would affect the breast cancer patient survival rate using the Kaplan–Meier plotter. We found poorer overall survival for high vs. low *FOXA1* gene expression amongst patients with any breast cancer subtype (*n* = 2976) as well as with HR+ breast cancer (*n* = 2005) specifically (Figure 4a,b). These results demonstrate that *FOXA1* might affect T2D and hormone-receptor-positive breast cancer (HR+ BC).

### 2.4. FOXA1 Is Highly Expressed in Hormone-Receptor-Positive Breast Cancer (HR+ BC) Cell Lines and Controls the HR+ Breast Cancer Cell Proliferation and Metastasis

**Figure 5:**
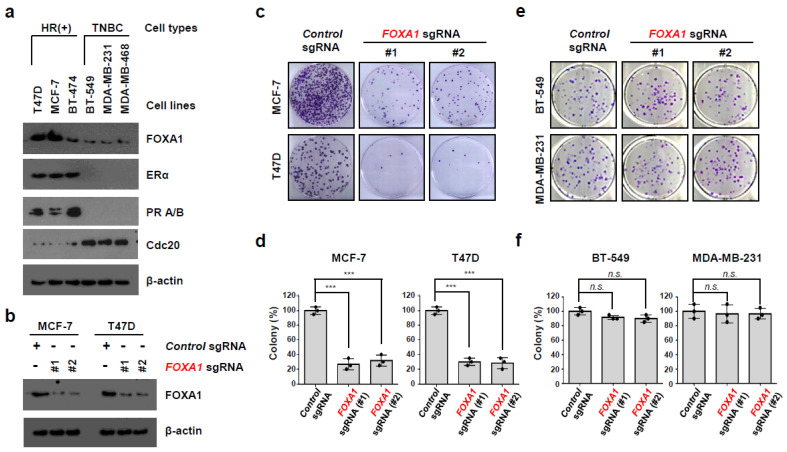
FOXA1 is highly amplified in hormone-receptor-positive breast cancer (HR+ BC) cells and regulates tumor cell growth. (**a**) Three hormonal breast cancer and three TNBC cell lines were analyzed by immunoblotting for the indicated proteins. ERα, estrogen receptor α; PR A/B, progesterone receptor A/B. (**b**) MCF-7 or T47D hormonal breast cancer cells were infected with the indicated plasmids (*Control* sgRNA or *FOXA1* sgRNA) and then were collected for immunoblot analysis for indicated proteins. (**c**–**f**) MCF-7, T47D, BT-549, and MDA-MB-231 cells were infected with the indicated plasmids (*Control* sgRNA or *FOXA1* sgRNA) and were plated for colony-forming assay (**c**,**e**). The results represent the means (±SE) of three independent experiments performed in triplicate. *** *p* < 0.001; n.s., non-significant. Statistical comparisons of scatter plots and bar graphs were performed by ANOVA with a multiple-comparisons test. The relevant data of (**d**,**f**) can be found in the Supplementary Materials.

**Figure 6:**
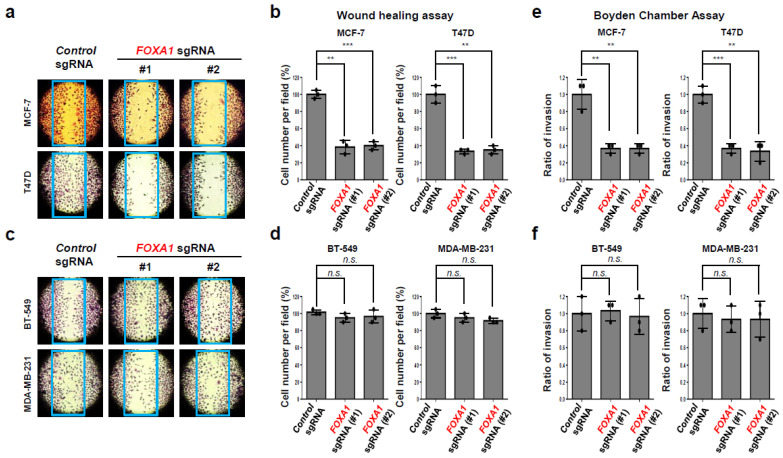
Loss of *FOXA1* prevents the metastasis of hormone-receptor-positive breast cancer (HR+ BC) cells. (**a**–**d**) MCF-7, T47D, BT-549, and MDA-MB-231 cells were infected with the indicated constructs (Control sgRNA or FOXA1 sgRNA). Cell lines cultured at 80–90% confluency were scratched with a 200 μL tip, washed thoroughly with 1× PBS, and cultured for 24 h in serum-free conditions. The cells were then re-cultured in 10% serum medium for 24 h, stained with 0.2% crystal violet, and observed under an inverted microscope. The blue box indicates day 0 when the cell wound healing assay was performed. The results represent the means (±SE) of three independent experiments performed in triplicate. ** *p* < 0.01, *** *p* < 0.001; n.s., non-significant. Statistical comparisons of scatter plots and bar graphs were performed by ANOVA with a multiple-comparisons test. (**e**,**f**) MCF-7, T47D, BT-549, and MDA-MB-231 cells were infected with the indicated constructs (*Control* sgRNA or *FOXA1* sgRNA) and cultured in a serum-free Boyden chamber for 24 h. The cells were incubated in the upper chamber, which contained Matrigel, in serum-free medium for 24 h. Subsequently, Matrigel penetration was assessed by introducing 20% FBS medium into the lower chamber. Visualization of cell penetration was achieved through staining with 0.2% Crystal Violet. Quantification of cell numbers of results represents the means (±S.E.) of three independent experiments performed in triplicate. ** *p* < 0.01, *** *p* < 0.001; n.s., non-significant. Statistical comparisons of scatter plots and bar graphs were performed by ANOVA with a multiple-comparisons test. The relevant data of (**b**,**d**–**f**) can be found in the Supplementary Materials.

*FOXA1* is a transcription factor that affects cancer initiation and promotion in several cancers, such as breast, endometrium, prostate, lung, and liver cancers [22,23,24,25]. As *FOXA1* was significantly expressed in hormone-receptor-positive breast cancer (HR+ BC) patients in Figure 2, Figure 3 and Figure 4, we confirmed the FOXA1 protein level in three different hormone-receptor-positive breast cancer (HR+ BC) cell lines (T47D, MCF-7, and BT-474) and three different TNBC cell lines (BT-549, MDA-MB-231, and MDA-MB-468). FOXA1 protein was highly expressed in only hormone-receptor-positive breast cancer (HR+ BC) cell lines but not in the TNBC cell lines (Figure 5a). ERα and PR A/B protein were highly expressed in the hormone-receptor-positive breast cancer (HR+ BC) cell lines; however, cell division cycle 20 (CDC20) was only detected in the TNBC cell lines [26,28]. In a previous study, we proved the high expression of CDC20 protein in TNBC cell lines and its role in TNBC cell proliferation and invasion [28]. To check our hypothesis that *FOXA1* would affect the hormone-receptor-positive breast cancer (HR+ BC) cell growth, we used CRISPR/Cas9 to knockout *FOXA1* (Figure 5b). We confirmed the *FOXA1* knockout using two different *FOXA1* sgRNA and found that it was targeted in MCF-7 and T47D (Figure 5b). Loss of *FOXA1* significantly decreased the tumor cell proliferation in MCF-7 (Figure 5c,d). These results were also found in another hormone-receptor-positive breast cancer (HR+ BC) cell line, T47D. However, the tumor cell growth inhibition effect caused by *FOXA1* gene deletion was not seen in TNBC cell lines, another breast cancer subtype (Figure 5e,f). These data indicate that *FOXA1* positively regulates tumor cell growth, specifically only in hormone-receptor-positive breast cancer (HR+ BC). To investigate if *FOXA1* might also affect the HR+ cancer cell movement and metastasis, we studied its effect through wound healing and the Boyden chamber assays (Figure 6). Deletion of the *FOXA1* gene resulted in a dramatic loss of tumor cell migration ability in the hormone-receptor-positive breast cancer (HR+ BC) cell lines MCF-7 and T47D (Figure 6a,b). However, this effect of reducing tumor cell migration due to loss of FOXA1 was not observed in the TNBC cell lines, such as BT-549 and MDA-MB-231 (Figure 6c,d). Furthermore, in tumor cell invasion experiments using Boyden chambers, the absence of the *FOXA1* gene had a significant effect on inhibiting metastasis of breast cancer-positive cell lines (Figure 6e,f) but no such effect in TNBC cell lines. These results indicate that the *FOXA1* gene is a factor that closely regulates not only tumor cell proliferation but also cancer metastasis in only hormone-receptor-positive breast cancer (HR+ BC) but not TNBC.

### 2.5. Metformin and FOXA1 Deletion Enhance Tamoxifen-Mediated Tumor Cell Growth Inhibition in Hormone-Receptor-Positive Breast Cancer (HR+ BC) Cells

**Figure 7:**
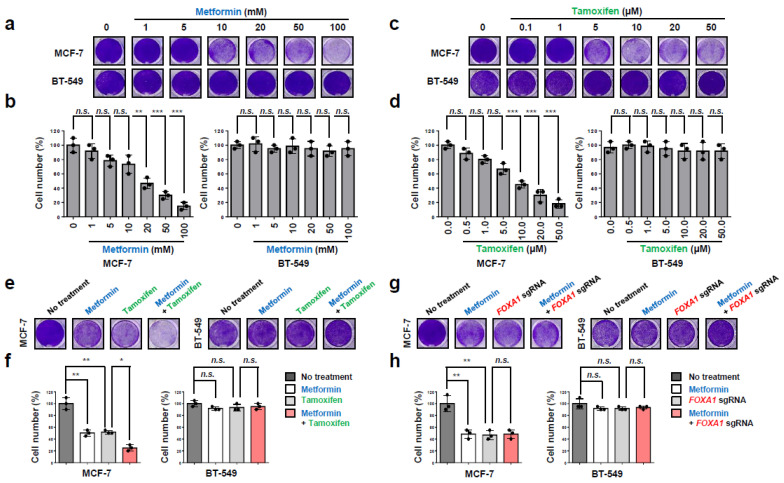
Metformin and *FOXA1* deletion enhance tamoxifen-mediated tumor growth inhibition in hormone-receptor-positive breast cancer (HR+ BC) cells. (**a**–**d**) MCF-7 and BT-549 cells were treated with metformin at different concentrations (0, 1, 5, 10, 20, 50, and 100 mM) or tamoxifen at different concentrations (0, 0.1, 1, 5, 10, 20, and 50 μM). After 3 days, the cell viability was measured. Representative image of cells stained with 0.1% crystal violet. The results represent the means (±SE) of three independent experiments performed in triplicate. ** *p* < 0.01, *** *p* < 0.001; n.s., non-significant. Statistical comparisons of scatter plots and bar graphs were performed by repeated-measures ANOVA with a multiple-comparisons test. (**e**–**h**) MCF-7 and BT-549 cells were treated with metformin (20 mM) or tamoxifen (10 μM) or were infected with *FOXA1* sgRNA. After 3 days, the cell viability was measured. Representative image of cells stained with 0.1% crystal violet. The results represent the means (±SE) of three independent experiments performed in triplicate. * *p* < 0.05, ** *p* < 0.01; n.s., non-significant. Statistical comparisons of scatter plots and bar graphs were performed by ANOVA with a multiple-comparisons test. The relevant data of (**b**,**d**,**f**,**h**) can be found in the Supplementary Materials.

Although tamoxifen is a widely used hormone-receptor-positive breast cancer (HR+ BC) specific therapy, the high dose leads to severe side effects and the discontinuation of the treatment in some cases [10,14]. We speculated that metformin or deletion of the *FOXA1* gene might contribute to a greater effect with a lower dose of tamoxifen in blocking hormone-receptor-positive breast cancer (HR+ BC) cell proliferation. Metformin was treated in a dose-dependent manner (0, 1, 5, 10, 20, 50, and 100 mM) in MCF-7 for 3 days to determine the optimal dosage. The MCF-7 cells were treated at 20 mM (IC_50_), which indicates that metformin prevents tumor cell growth (Figure 7a,b). This effect of metformin was not observed in the TNBC BT-549 cell line. We also treated MCF-7 cells with tamoxifen in a dose-dependent manner (0, 0.1, 1, 5, 10, 20, and 50 µM) and found the IC_50_ value of 10 µM (Figure 7c,d). However, no such effect was observed in the BT-549 cell line of TNBC, which is not a hormone-positive cell line. Furthermore, we checked the effectiveness of the combination of metformin and tamoxifen in inhibiting hormone-receptor-positive breast cancer (HR+ BC) cell growth (Figure 7e,f). In the metformin-treated group, the cancer cell proliferation decreased by around 50% in comparison to the no-treatment group (control). Likewise, the tamoxifen-treated group also had 50% of the cell number to the control. Surprisingly, the metformin and tamoxifen combination significantly blocked cell growth by 50% more than the individual treatments in MCF-7 hormone-receptor-positive breast cancer (HR+ BC) cell lines (Figure 7e,f). Meanwhile, in BT-549, a TNBC cell line, no synergistic effect was found in inhibiting cell proliferation by treatment with metformin alone or tamoxifen alone, as well as the combination of these two drugs (Figure 7e,f). These results confirm the synergic sensitivity of combining metformin and tamoxifen in inhibiting tumor cell growth in hormone-receptor-positive breast cancer (HR+ BC) cells but not TNBC. Meanwhile, *FOXA1* KO and metformin combination had no change compared to the individual treatments, suggesting that *FOXA1* is regulated by metformin signals (Figure 7g,h). Additionally, the TNBC cell line BT-549 was used as a negative control in this experiment to verify the experimental process. Accumulatively, metformin plays an important role in decreasing the tumor cell growth in hormone-receptor-positive breast cancer (HR+ BC) with tamoxifen hormonal therapy.

### 2.6. Metformin Is Effective in Treating Tamoxifen-Treated Hormone-Receptor-Positive Breast Cancer (HR+ BC) Cells in an Ex Vivo Model

**Figure 8:**
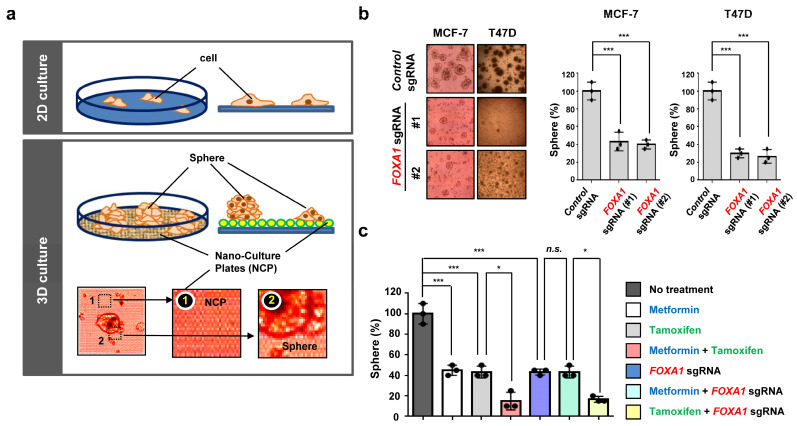
The combination of metformin and tamoxifen has a synergistic effect in inhibiting the growth of hormone-receptor-positive breast cancer (HR+ BC) ex vivo. (**a**) A schematic diagram of the difference between 2D and 3D cell cultures. NCPs, nano-culture plates; sphere, a form in which cancer cells grow in the form of a mass. (**b**) MCF-7 and T47D cells were infected with the indicated constructs (*Control* sgRNA or *FOXA1* sgRNA), and then cells were plated at low density (1000 cells) into 60 mm cell culture plates and were treated with the indicated chemicals for three days in 3D cell culture. Quantification of cell numbers of results represents the means (±S.E.) of three independent experiments performed in triplicate; *** *p* < 0.001. Statistical comparisons of scatter plots and bar graphs were performed by ANOVA with a multiple-comparisons test. Scale bar, 100 μm. (**c**) MCF-7 cells were treated with metformin (20 mM) or tamoxifen (10 μM) or were infected with *FOXA1* sgRNA. After 24 h, cells were plated at low density (1000 cells) into 60 mm cell culture plates and were treated with the indicated chemicals for three days in 3D cell culture. The results represent the means (±SE) of three independent experiments performed in triplicate. * *p* < 0.05, *** *p* < 0.001; n.s., non-significant. Statistical comparisons of scatter plots and bar graphs were performed by ANOVA with a multiple-comparisons test. The relevant data of (**b**,**c**) can be found in the Supplementary Materials.

We examined the tumor cell growth in hormone-receptor-positive breast cancer (HR+ BC) after seeding the nano-culture plates for the 3D organoid (ex vivo) culture system [26,27,28,44]. The 3D organoid culture system is an advanced experimental method to check drug effects [26,44,45]. It is commonly used as the cancer cells grow in a multi-layer and spheroid formation compared to the monolayer in 2D culture (Figure 8a). Furthermore, the ex vivo model has a high genetic accuracy compared to the animal models [26,44]. The hormone-receptor-positive breast cancer (HR+ BC) cell growth rate is quantified by counting the formation of spheroids in the 3D organoid system [45]. While general tumor spheroid formation occurred in the *control* sgRNA, the cancer spheroid formation could not be as well detected in the two different *FOXA1* deletion groups for hormone-receptor-positive breast cancer (HR+ BC), such as MCF-7 and T47D cell lines (Figure 8b). We further investigated the metformin effect with tamoxifen, as demonstrated in Figure 7c, through the ex vivo model. The combination of metformin and tamoxifen had a synergic effect on decreasing cancer spheroid formation in hormone-receptor-positive breast cancer (HR+ BC) (Figure 8c). Likewise, tamoxifen and *FOXA1* sgRNA also had similar effects but not metformin and *FOXA1* KO (Figure 8c). Overall, these results demonstrate that metformin and *FOXA1* deletion assists the tamoxifen-mediated cancer spheroid formation inhibition in hormone-receptor-positive breast cancer (HR+ BC) cells.

## 3. Discussion

In this study, we found that T2D had a close relationship with only the hormone-positive (HR+) breast cancer subtype through the 53,805 genes dataset from NCBI GEO (Figure 1). In a previous study, we observed the clue that genes highly expressed in only HR+ BC are related to metabolism, neuronal, and cell proliferation [26,27,28]. In this large data set, we found 3728 genes were associated with hormone-receptor-positive breast cancer (HR+ BC) and 1470 distinct genes found in TNBC. Of these, only 1150 genes exhibited concurrent expression in both breast cancer subtypes, highlighting their potential impact on distinguishing hormone-receptor-positive breast cancer (HR+ BC) from triple-negative breast cancer (TNBC) [26,27,28]. As a result of grouping these genes, it was found that many genes related to cell metabolism, nerve-related, and cell proliferation were expressed in hormone-receptor-positive breast cancer (HR+ BC), whereas cancer metastasis and inflammation-related genes were overexpressed in TNBC. Therefore, since diabetes is a metabolic disorder, it is reasonable to investigate the correlation between T2D and HR+ BC. We obtained 26 genes with high expression in all three cell lines from the hormone-receptor-positive breast cancer (HR+ BC) group and reviewed whether their genetic values were related to diabetes through several research papers. As a result, we were able to find a very close relationship with diabetes in 14 genes [29,30,31,32,33,34,35,36,37,38,39,40,41,42,43]. Five of the 14 diabetes-related genes, *FOXA1*, *KIF22*, *PAK4*, *MTA3*, and *FGFR3*, were specifically highly expressed in only the ER+/PR+ and ER+/PR− subtypes but not ER−/PR+ and ER−/PR− through an analysis using 4032 patient tissue samples from Breast Cancer Gene Expression Miner v4.5 (Figure 2). This result confirmed our finding that the genetic underpinnings of T2D are linked to hormone-receptor-positive breast cancer (HR+ BC) (Figure 1).

Our specific focus was on *FOXA1* due to its elevated expression among diabetes-related genes within the hormone-receptor-positive breast cancer (HR+ BC) subtype, as determined by gene expression analysis of breast cancer patient tissue samples. Furthermore, FOXA1’s documented role in type 2 diabetes (T2D) and its expression in breast cancer has been extensively reported in scientific literature [24,38]. High expression of the *FOXA1* gene was associated with worse overall survival in 2005 ER+/PR+ breast cancer patients from Kaplan–Meier plotter (Figure 4). In our in vitro experiment, we observed FOXA1 expression exclusively in the three hormone-receptor-positive breast cancer (HR+ BC) cell lines, with no detection in the triple-negative breast cancer (TNBC) cell lines. Some papers reported that FOXA1 is highly expressed in ER-breast cancers [46], but we were not able to find this in the BT-549, MDA-MB-231, and MDA-MB-468 TNBC cell lines. To study the *FOXA1* gene function, we completed a CRISPR/Cas9 gene KO in HR+ breast cancer cell lines. The cell proliferation in hormone-receptor-positive breast cancer (HR+ BC) cell lines MCF-7 and T47D was significantly decreased with the loss of *FOXA1* (Figure 5). Additionally, the migration and invasion of the hormone-receptor-positive breast cancer (HR+ BC) cell lines were also significantly decreased with *FOXA1* KO (Figure 6). These data indicate that the diabetes-related gene *FOXA1* plays a critical role in regulating cancer cell growth and metastasis in hormone-receptor-positive breast cancer (HR+ BC). However, uncertainty remains regarding which genes regulate the *FOXA1* gene and which proteins are regulated in HR+ BC.

Tamoxifen is an effective hormonal therapy for hormone-receptor-positive breast cancer (HR+ BC) patients as it decreases the recurrence rate by 50% and the mortality rate by a third with five years of adjuvant endocrine therapy [1,3,4,5,10,13,14]. Also, the extension of the therapy for another five years has been proven to affect recurrence risks up to 40%. However, nonadherence to endocrine therapy is common, and previous studies have reported poorer survival outcomes because of nonadherence, especially for younger HR+ breast cancer patients [14,47]. Furthermore, tamoxifen can cause hot flashes, uterine cancer, blood clots, gastrointestinal hypersensitivity, headache, and dizziness [13], which can contribute to early treatment cessation [1,5,48]. Therefore, an approach to lower the dosage of the tamoxifen treatment while receiving the benefits of decreased side effects and maximizing hormonal therapy effects could be advantageous. The use of metformin in combination with tamoxifen in the hormone-receptor-positive breast cancer (HR+ BC) subtype may be one such possible opportunity. Surprisingly, the combination had a synergic effect in significantly decreasing the tumor cell growth of hormone-receptor-positive breast cancer (HR+ BC) in comparison with each individual treatment (Figure 7). These data suggest a novel drug combination strategy for hormone-receptor-positive breast cancer (HR+ BC) treatment that could provide additional benefits. Recent clinical data reported that metformin did not significantly affect overall survival or distant recurrence-free survival in HR+ breast cancer [49]. However, the results of this study were based on metformin treatment in female breast cancer patients without diabetes, and given that most of the study subjects were mainly white, non-Hispanic, and North American people, it may not have provided an appropriate treatment group to test the beneficial effects of metformin. Additional research related to this effect seems necessary.

**Figure 9:**
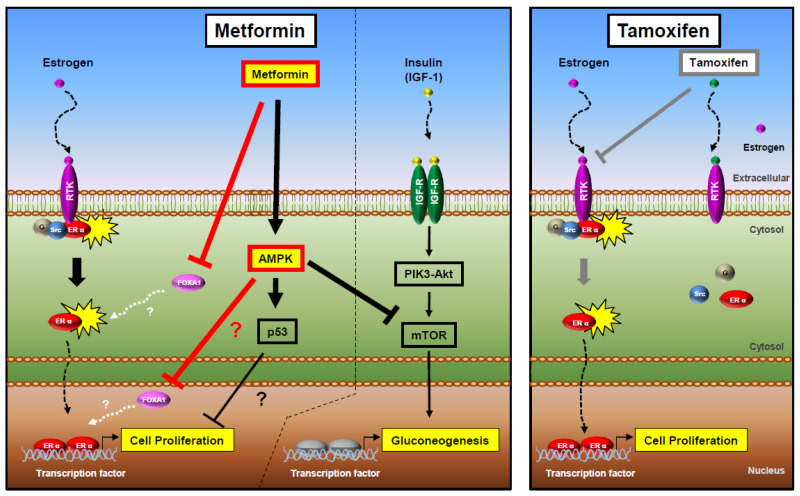
*FOXA1* is negatively regulated by metformin in HR+ breast cancer (BC). The schematic diagram illustrates the signaling pathways of metformin and tamoxifen. Estrogen activates receptor tyrosine kinases (RTKs) to bind to membrane ERα and G proteins, resulting in an increase in second messengers and the activation of protein kinase cascades such as the MAPK and PI3K/Akt signaling pathways. Metformin phosphorylates AMPK, thereby inhibiting cell proliferation through p53 regulation. Additionally, AMPK activation by metformin suppresses the insulin-induced PI3K pathway via mTOR signaling. *FOXA1*, which promotes transcription for cell proliferation in HR+ BC, is negatively regulated by metformin. Meanwhile, tamoxifen competitively binds to estrogen receptor tyrosine kinase (RTK) in HR+ BC, preventing ERα from phosphorylating second messengers. This action blocks ERα translocation to DNA promoter regions, thereby inhibiting tumor cell proliferation. The antidiabetic drug metformin may exert a potential synergistic effect in patients with hormone-receptor-positive breast cancer when combined with tamoxifen, a hormone therapy.

The AMPK has an important role in cellular and organismal metabolism and is linked to cell death through metformin. AMPK comprises a catalytic α subunit and regulatory β and γ subunits. It is an energy-sensing protein that is activated by the phosphorylation of upstream kinases at the threonine residue (Thr-172) in response to cellular stresses such as glucose deprivation, oxidative stress, hyperosmotic stress, hypoxia, and tissue ischemia [47,48,50,51]. AMPK also inhibits the mTOR pathway, which plays a vital role in regulating cell growth. mTOR regulates various downstream proteins, such as p70S6K, which controls transcription and translation [52]. Meanwhile, metformin has an anti-tumor effect by activating the AMPK pathway and mTOR inhibition [53]. It was previously reported that metformin inhibits invasion and metastasis development through AMPK/p53 axis activation in melanoma [54]. We found that metformin decreases tumor growth in HR+ breast cancer through the downregulation of *FOXA1*. FOXA1 is a transcription factor that affects organogenesis and cancer progression in various cancers such as breast, prostate, endometrium, liver, and lung cancers [22,23,24,25]. Specifically, in breast cancer, FOXA1 was reported to regulate the ERα function in hormone-receptor-positive breast cancer (HR+ BC) [55], but the exact mechanism is unclear. In the clinical database results, we checked the high expression of *FOXA1* in only the hormone-receptor-positive breast cancer (HR+ BC) subtype and the worse overall survival for high *FOXA1* gene expression in ER + /PR+ breast cancer patients. Also, the *FOXA1* KO significantly decreased the tumor cell growth and invasion in hormone-positive (HR+) breast cancer cell lines, MCF-7 and T47D, but not TNBC. When the hormone-receptor-positive breast cancer (HR+ BC) cells were treated with metformin, the cancer cell group with *FOXA1* KO did not affect cell growth, indicating that *FOXA1* is regulated by metformin. From here, we deduced that *FOXA1* is related to AMPK, but whether the relationship is direct or indirect is a further study (Figure 9). A recent study by Lundgren et al. explored the predictive value of gene expression (GEX) for the efficacy of tamoxifen in 236 premenopausal breast cancer patients with early-stage ER+/HER2− tumors [56]. Interestingly, they found that low expression of the *FOXA1* genes was associated with an improved response to tamoxifen treatment. These findings suggest an inverse correlation between the efficacy of tamoxifen and the GEX level of *FOXA1*, indicating that the therapeutic efficacy of tamoxifen is significantly influenced by the expression level of the *FOXA1* gene in patients with ER+/HER2− tumors. Figure 8c from our study demonstrated that the combined treatment of tamoxifen and *FOXA1* gene deletion exhibited significantly higher anticancer efficacy than tamoxifen alone. This study is particularly valuable as it suggests that combination therapy with tamoxifen and FOXA1 gene-targeting therapy may serve as an effective anticancer strategy, even in patients with hormone-positive breast cancer with high FOXA1 expression.

To confirm our results, we used the 3D organoid culture system. Other experimental methods, such as the 2D cell culture and animal models, each have their shortcomings. The 2D cell culture only allows cells to grow in a monolayer form, which decreases the clinical relevance and drug dosage accuracy [26,44,45]. The animal model has a high cost and is very time-consuming. Furthermore, when cancer cells are administered to the animals, the immune system of the animals is activated, which either modifies the cancer cells or destroys the cancer cells in some cases [45]. However, the 3D organoid culture system is an advanced method of experimentation because of its low cost, time efficiency, high clinical relevance, and high clinical relevance. Moreover, the cancer cells in the 3D organoid model grow in a multilayer, spheroid formation, which is highly accurate to human cancer cells in the body [26,44,45]. We found that the deletion of the *FOXA1* gene decreased the cancer cell spheroid formation in the hormone-receptor-positive breast cancer (HR+ BC) cell lines using 3D ex vivo. Interestingly, we also confirmed the synergic effect of the combination of tamoxifen and metformin in significantly reducing the tumor cell growth of hormone-receptor-positive breast cancer (HR+ BC) cell lines. These results suggest that metformin can be a good tool and could potentially reduce the side effects of hormonal treatment for HR+ breast cancer with tamoxifen.

## 4. Materials and Methods

### 4.1. Cancer Data Collection and Processing

By using cancer datasets containing gene expressions and patients’ clinical information from the Gene Expression Omnibus (http://www.ncbi.nlm.nih.gov/geo, accessed on 15 December 2018), we studied the gene expressions in six different breast cancer cell lines [26,27,28]. A classification rule was employed that summarized the standardized levels of gene expression. First, the diabetes-related genes and β-actin were selected from NCBI data. The variability of gene expression in human HR+ BC cell lines was calculated by using other independent research groups that compared β-actin as the most stable housekeeping gene for normalizing gene expression. Each value was then divided by the average of all values. Finally, log2-transformed expression (or concentration) values were used. Statistics were analyzed by the GraphPad Prism software (version 6.0). To investigate the diabetes-related genes expressions in different breast cancer subtypes such as ER+/PR+, ER+/PR−, ER−/PR+, and ER−/PR−, we used the Breast Cancer Gene-Expression Miner v4.5 (which is now v5) (http://bcgenex.ico.unicancer.fr/BC-GEM/GEM-Accueil.php?js=1, accessed on 11 January 2021). The gene expressions of 14 diabetes-related genes were searched for in various breast cancer subtypes from 4032 breast cancer patient tissue samples. The gene correlations between *FOXA1* and diabetes-related genes and *FOXA1* and TNBC-related genes in the ER+/PR+ breast cancer subtype were assessed using the Breast Cancer Gene-Expression Miner v4.5 (http://bcgenex.ico.unicancer.fr/BC-GEM/GEM-Accueil.php?js=1, accessed on 11 January 2021). The overall survival rate for *FOXA1* in all breast cancer (*n* = 2976) and the ER+/PR+ specific subtype (*n* = 2005) was found using the Kaplan–Meier plotter (https://kmplot.com/analysis/index.php?p=service&cancer=breast_rnaseq_gse96058, accessed on 22 January 2021) with human breast cancer patient’s dataset. *p*-values were calculated using unpaired two-tailed Student’s *t*-tests.

### 4.2. Cells and Cell Lines, Reagents, and Gene Silencing 

All cell lines were sourced from commercial vendors such as ATCC (Manassas, VA, USA). Human breast cancer cell lines were cultured in RPMI (Thermo Fisher Scientific, Waltham, MA, USA) with 10% FBS. Hormone-receptor-positive breast cancer (HR+ BC) cell lines such as T47D, MCF-7, and BT-474 were cultured for the cell lines used in this study, and TNBC cell lines such as BT-549, MDA-MB-231, MDA-MB-468 were used as negative controls [26,27,28]. Metformin (PHR1064) and tamoxifen were obtained from Sigma-Aldrich (St. Louis, MO, USA). Each sample was prepared at a ratio of 1:1000 to facilitate drug administration in various ratios (metformin: 1, 5, 10, 20, 50, 100 M; tamoxifen: 0.1, 1, 5, 10, 20, 50 mM) according to each experimental situation. All samples were stored frozen at −20 °C before and after use. To knock out the *FOXA1* gene, we utilized the CRISPR/Cas9 system targeting exon 2 of FOXA1 using the *FOXA1* sgRNA plasmid (Lenti_gRNA-GFP (LRG), #105511). These plasmids were purchased from Addgene (Watertown, MA, USA) or GenScript (Piscataway, NJ, USA), and the specific sgRNA sequences used are as follows: sgRNA No. 1: 5′-GACTATCATATGCTTACCGT-3′, sgRNA No. 2: 5′-GTTGGACGGCGCGTACGCCA-3′, sgRNA No. 3: 5′-TAGCTGCGCTTGAACGTCT-3′. Lentivirus production was performed in HEK293T cells transfected with lentiviral transfer vector and helper vector (psPAX2 and pVSV-G) using Lipofectamine 3000 (Thermo Fisher Scientific, Dreieich, Germany). Lentiviral supernatants were collected 48 h after transfection, immediately frozen, and stored at −80 °C. To determine the appropriate titer for lentivirus infection, MCF-7 cells were infected with two-fold serial dilutions of lentiviral supernatants. After infection, cells were selected with puromycin (2 µg/mL) for 3 days. Cell viability was assessed by counting the number of cells expressing GFP under a fluorescence microscope to determine the optimal multiplicity of infection (MOI = 0.3). On day 3 after lentivirus infection, infected cells were harvested as day 0 samples or reselected with 2 µg/mL puromycin for continued culture. *FOXA1* gene knockout was confirmed by FOXA1 protein expression using Western blot.

### 4.3. Western Blot and Antibodies

Protein lysate samples from various breast cancer cell lines were lysed using RIPA lysis buffer supplemented with protease inhibitors, including with 1 mM PMSF on ice. The protein lysates were initially separated via gel electrophoresis and subsequently transferred onto PVDF membranes [26,27,28,44]. Following this, the membranes were blocked in TBS-T buffer with 5% fat-free milk for 1 h at room temperature. Then, the PVDF membrane was incubated with specific primary antibodies. Rabbit polyclonal antibodies recognizing FOXA1 (#58613), Progesterone Receptor A/B (#3176), and Estrogen Receptor alpha (ab75635) were purchased from Cell Signaling and Abcam. Mouse monoclonal antibody recognizing Cdc20 (sc-5296) was purchased from Santa Cruz Biotechnology. Anti-β-actin mouse antibody was purchased from Sigma. The membrane was washed three times with 1% TBST and subsequently incubated for 1 h with rabbit anti-mouse IgG-HRP secondary antibodies (Abcam, Cambridge, UK) as described previously [26,27,28,44].

### 4.4. Colony-Forming Assay

MCF-7, T47D, BT-549, and MDA-MB-231 cells were plated at low density (500–1000 cells) into 60 mm cell culture plates. These cells were then either infected with *FOXA1* sgRNA or treated with metformin and tamoxifen. For colony formation assay [27,28], once sufficient colonies were visible, typically after 2–3 weeks, cells were washed twice with PBS, fixed in ice-cold 70% methanol for 30 min, and then stained with 0.2% Crystal violet for 2–3 h. The following day, cells were rinsed with PBS and air-dried. Stained breast cancer cells were counted with a magnifying glass or a microscope.

### 4.5. Migration and Invasion Assays

For the migration assay (wound-healing assay) [26,27,28], MCF-7, T47D, BT-549, and MDA-MB-231 cells were cultured in 10 cm cell culture dishes and subjected to either control or *FOXA1* sgRNA infection for 24 h. Subsequently, the cells were seeded in 6-well plates to achieve 80–90% confluence and cultured until confluent (approximately 3 days). Upon reaching confluence, the cells were treated with serum-free medium supplemented with 10% FBS and cultured for 24 h. Prior to serum-free medium replacement, thorough washing with 1× PBS and subsequent aspiration were performed to ensure complete removal of residual serum from the cell culture dish. Confluent monolayer cells were then gently scraped from each well (3 rows/well) using a P200 tip, with floating cells in the medium removed by washing with 1× PBS and aspiration. Subsequently, the cells were replenished with complete medium containing 10% FBS. After 24 h, the cells were stained with 0.2% Crystal Violet solution containing 10% methanol, followed by observation under an inverted microscope, and the migrated cells were captured via photography. Quantification of cell migration involved counting the cells within the scratch area (designated as time 0) across different regions of the wound.

For the Matrigel invasion assay, cells in 10 cm cell culture dishes were infected with either control or *FOXA1* sgRNA for 24 h. After 8 h, the medium was replaced with serum-free medium and cultured until reaching approximately 80% confluence. Subsequently, the cells were seeded into 24-well invasion chambers (Corning, New York, NY, USA, 354480). The chambers were interconnected for 24 h to allow cells cultured in serum-free medium in the upper chamber, containing Matrigel, to migrate towards the complete medium containing 20% FBS in the lower chamber. Cells embedded within Matrigel were excluded from the experimental analysis, and only cells that migrated out of Matrigel were enumerated. Each sample was plated in triplicate (500,000 cells/insert), adhering strictly to the recommended standard protocol. The measurement of cell invasion involved staining the filters with 0.2% Crystal Violet, followed by enumeration of invading cells. 

### 4.6. Three-Dimensional Organoid Assay

For 3D organoid assays, MCF-7 or T47D human hormone-receptor-positive breast cancer (HR+ BC) cell lines were cultured on NanoCulture plates (SCIVAX) [26,27,28,44]. Before conducting the experiment, the growth characteristics of breast cancer are identified in advance by confirming the spherical proliferation rate and size of breast cancer cells in advance. After seeding cells, cells were treated with tamoxifen and metformin or were infected with Control sgRNA or *FOXA1* sgRNA. Then, cell spheroid images were observed and counted by microscope.

### 4.7. Statistical Analyses

For each in vitro or ex vivo experiment, three independent replicates were assessed, and the combined data were presented as mean ± standard error. The results were presented as individual data points or as mean ± standard deviation (S.D.). Statistical analyses were conducted using GraphPad Prism software (version 6.0; GraphPad Software, San Diego, CA, USA). Comparisons of scatter plots and bar graphs were performed using either one-way analysis of variance (ANOVA) or two-way ANOVA for multiple comparisons, followed by the Holm–Sidak post hoc test. A *p*-value of <0.05 was considered significant. Statistical significance thresholds were defined as *p* < 0.05 (*), *p* < 0.01 (**), *p* < 0.001 (***), and *p* < 0.0001 (****); n.s., not significant.

## 5. Conclusions

In summary, the diabetes-related gene *FOXA1* may play an important role in controlling the cell growth and invasion of hormone-receptor-positive breast cancer (HR+ BC). The diabetic medication metformin might be a potential synergistic treatment for HR+ breast cancer with tamoxifen. It may also aid in decreasing the side effects of tamoxifen.

## Data Availability

The datasets used and/or analyzed during this study are available from the corresponding author upon reasonable request.

## Supplementary Materials

The following supporting information can be downloaded at https://www.mdpi.com/article/10.3390/ijms25137494/s1.

## Abbreviations

ER, estrogen; PR, progesterone; HR+ breast cancer, hormone-receptor-positive breast cancer; KO, knockout; FOXA1, forkhead box A1; 3D, three-dimensional; metformin, 1,1-dimethylbiguanide hydrochloride; TNBC, triple-negative breast cancer.
