# Supplementary material for: Metformin Prevents Tumor Cell Growth and Invasion of Human Hormone Receptor-Positive Breast Cancer (HR+ BC) Cells via FOXA1 Inhibition
Source: Int J Mol Sci. 2024 Jul 8;25(13):7494. doi: 10.3390/ijms25137494 (PMC11242876; doi:10.3390/ijms25137494)
Supplement: Supplementary file 1 [file ijms-25-07494-s001.zip › ijms-3068137-supplementary.pdf]

Colony forming

MCF-7								
Control sgRNA			*FOXA1* sgRNA (#1)			*FOXA1* sgRNA (#2)		
a	b	c	a	b	c	a	b	c
100	105	95	35	25	20	40	25	30

T47D								
Control sgRNA			*FOXA1* sgRNA (#1)			*FOXA1* sgRNA (#2)		
a	b	c	a	b	c	a	b	c
100	105	95	25	30	35	20	35	30

BT-549								
Control sgRNA			*FOXA1* sgRNA (#1)			*FOXA1* sgRNA (#2)		
a	b	c	a	b	c	a	b	c
100	105	95	90	95	90	90	85	95

MDA-MB-231								
Control sgRNA			*FOXA1* sgRNA (#1)			*FOXA1* sgRNA (#2)		
a	b	c	a	b	c	a	b	c
100	90	110	95	85	110	95	105	90

Wond healing

MCF-7								
Control sgRNA			*FOXA1* sgRNA (#1)			*FOXA1* sgRNA (#2)		
a	b	c	a	b	c	a	b	c
100	105	95	40	30	45	35	45	40

T47D								
Control sgRNA			*FOXA1* sgRNA (#1)			*FOXA1* sgRNA (#2)		
a	b	c	a	b	c	a	b	c
100	110	90	30	35	35	40	30	35

BT-549								
Control sgRNA			*FOXA1* sgRNA (#1)			*FOXA1* sgRNA (#2)		
a	b	c	a	b	c	a	b	c
100	105	100	90	100	95	90	95	105

MDA-MB-231								
Control sgRNA			*FOXA1* sgRNA (#1)			*FOXA1* sgRNA (#2)		
a	b	c	a	b	c	a	b	c
100	95	105	95	100	90	90	95	90

Boyden chamber

MCF-7								
Control sgRNA			*FOXA1* sgRNA (#1)			*FOXA1* sgRNA (#2)		
a	b	c	a	b	c	a	b	c
1	1.2	0.8	0.3	0.4	0.4	0.4	0.3	0.4

T47D								
Control sgRNA			*FOXA1* sgRNA (#1)			*FOXA1* sgRNA (#2)		
a	b	c	a	b	c	a	b	c
1	0.9	1.1	0.4	0.3	0.4	0.4	0.4	0.2

BT-549								
Control sgRNA			*FOXA1* sgRNA (#1)			*FOXA1* sgRNA (#2)		
a	b	c	a	b	c	a	b	c
1	0.8	1.2	1.1	1.1	0.9	0.8	1.2	0.9

MDA-MB-231								
Control sgRNA			*FOXA1* sgRNA (#1)			*FOXA1* sgRNA (#2)		
a	b	c	a	b	c	a	b	c
1	0.8	1.2	0.9	1.1	0.8	1.1	1	0.7

cell number
(tumer cell growth)

Metformin		0			1			5			10			20			50			100 (mM)		
	MCF-7	a	b	c	a	b	c	a	b	c	a	b	c	a	b	c	a	b	c	a	b	c
		100	110	90	100	95	80	85	80	70	75	85	60	55	45	40	35	30	25	20	10	15

Metformin		0			1			5			10			20			50			100 (mM)		
	BT-549	a	b	c	a	b	c	a	b	c	a	b	c	a	b	c	a	b	c	a	b	c
		100	105	95	90	110	105	95	90	100	90	110	95	95	105	85	100	90	85	95	85	105

Tamoxifen		0			0.1			1			5			10			20			50 (uM)		
	MCF-7	a	b	c	a	b	c	a	b	c	a	b	c	a	b	c	a	b	c	a	b	c
		100	95	105	95	80	90	75	80	85	75	65	60	45	40	50	35	35	20	25	15	15

Tamoxifen		0			0.1			1			5			10			20			50 (uM)		
	BT-549	a	b	c	a	b	c	a	b	c	a	b	c	a	b	c	a	b	c	a	b	c
		100	95	95	105	95	100	90	105	100	95	105	85	95	100	80	80	90	105	100	80	95

	Control			Metformin			Tamoxifen			M+T		
MCF-7	a	b	c	a	b	c	a	b	c	a	b	c
	100	110	90	55	45	50	55	50	50	25	30	20

	Control			Metformin			Tamoxifen			M+T		
BT-549	a	b	c	a	b	c	a	b	c	a	b	c
	100	105	95	90	95	90	90	100	90	90	100	95

	Control			Metformin			FOXA1 sgRNA (#1)			M+F		
MCF-7	a	b	c	a	b	c	a	b	c	a	b	c
	100	90	110	55	40	50	55	40	45	40	55	50

	Control			Metformin			FOXA1 sgRNA (#1)			M+F		
BT-549	a	b	c	a	b	c	a	b	c	a	b	c
	100	100	110	90	90	95	95	90	90	95	90	95

Sphere form

MCF-7								
Control sgRNA			*FOXA1* sgRNA (#1)			*FOXA1* sgRNA (#2)		
a	b	c	a	b	c	a	b	c
100	110	90	55	35	40	35	40	45

T47D								
Control sgRNA			*FOXA1* sgRNA (#1)			*FOXA1* sgRNA (#2)		
a	b	c	a	b	c	a	b	c
100	110	90	30	35	25	35	25	20

Control			Metformin			Tamoxifen			M+T			FOXA1 sgRNA (#1)			M+F			T+F		
a	b	c	a	b	c	a	b	c	a	b	c	a	b	c	a	b	c	a	b	c
100	110	90	40	50	45	50	40	40	10	25	10	45	40	45	40	50	40	20	15	15
